# The Role of Familiarity for Representations in Norm-Based Face Space

**DOI:** 10.1371/journal.pone.0155380

**Published:** 2016-05-11

**Authors:** Stella J. Faerber, Jürgen M. Kaufmann, Helmut Leder, Eva Maria Martin, Stefan R. Schweinberger

**Affiliations:** 1 DFG Research Unit Person Perception, Friedrich Schiller University of Jena, Jena, Germany; 2 Department of General Psychology and Cognitive Neuroscience, Friedrich Schiller University of Jena, Jena, Germany; 3 Department for Basic Psychological Research and Research Methods, University of Vienna, Vienna, Austria; 4 Michael Stifel Center Jena for Data-Driven and Simulation Science, Friedrich Schiller University of Jena, Jena, Germany; Bournemouth University, UNITED KINGDOM

## Abstract

According to the norm-based version of the multidimensional face space model (nMDFS, Valentine, 1991), any given face and its corresponding anti-face (which deviates from the norm in exactly opposite direction as the original face) should be equidistant to a hypothetical prototype face (norm), such that by definition face and anti-face should bear the same level of perceived typicality. However, it has been argued that familiarity affects perceived typicality and that representations of familiar faces are qualitatively different (e.g., more robust and image-independent) from those for unfamiliar faces. Here we investigated the role of face familiarity for rated typicality, using two frequently used operationalisations of typicality (deviation-based: DEV), and distinctiveness (face in the crowd: FITC) for faces of celebrities and their corresponding anti-faces. We further assessed attractiveness, likeability and trustworthiness ratings of the stimuli, which are potentially related to typicality. For unfamiliar faces and their corresponding anti-faces, in line with the predictions of the nMDFS, our results demonstrate comparable levels of perceived typicality (DEV). In contrast, familiar faces were perceived much less typical than their anti-faces. Furthermore, familiar faces were rated higher than their anti-faces in distinctiveness, attractiveness, likability and trustworthiness. These findings suggest that familiarity strongly affects the distribution of facial representations in norm-based face space. Overall, our study suggests (1) that familiarity needs to be considered in studies of mental representations of faces, and (2) that familiarity, general distance-to-norm and more specific vector directions in face space make different and interactive contributions to different types of facial evaluations.

## Introduction

Models of face representation aim at elucidating face recognition and face classification processes and often use a framework that is based on the similarity between faces [1]. These models describe how stored face representations influence the encoding, classification and possible recognition of familiar or unfamiliar faces. According to the influential norm-based version of the multidimensional face space model (nMDFS, [2]), a face is encoded as a point in an n-dimensional space and its location is defined by a vector from the norm—i.e. the centre—to that point. The norm is the central tendency of all dimensions, which serve to discriminate faces. Those dimensions are explicitly not further specified, although they are assumed to correspond to physiognomic features such as hair colour and length, face shape or age ([2], p.166). This specific relation to the norm is critical for the nMDFS in that it assumes a gradual and concentric increase in perceived typicality (or decrease in perceived distinctiveness) the closer a face is located to the norm. Moreover, a common routine to approximate or operationalize the norm uses a digital average across a large number of faces (e.g., [3]). Accordingly, the typicality or distinctiveness within the nMDFS is defined by the *distance* of an individual face to that average.

Evidence for the nMDFS was provided by studies investigating contrastive aftereffects caused by adaptation, which demonstrated that adaptation to specific face images caused effects that were confined to a particular identity vector, or trajectory [3–7]. Note that within face space a face and its corresponding anti-face are on the same trajectory, which intersects the average face. Thus, an anti-face deviates from the norm in exactly opposite direction as their corresponding faces. For illustration refer to Faerber, Kaufmann, and Schweinberger [7]. Specifically, the pattern of adaptation effects to anti-faces provides strong evidence that the mental representations of a face and its anti-face are linked via a common trajectory through the norm face. This is because such adaptation effects were found to be restricted to this specific trajectory and are not due to the similarity or distance between faces [5, 8]. These seminal results strengthened the view that the processing of faces and their anti-faces involve norm-based neuronal representations, a hypothesis that received further support from a recent EEG study that combined anti-face adaptation and priming [7]. Although the perceptual interconnection of faces through trajectories in mental face space has been well established, there is a lack of direct empirical evidence on another prediction derived from the nMDFS model—that faces and their anti-faces, due to their equidistance to the norm, share equivalent levels of perceived typicality/distinctiveness.

Facial typicality and distinctiveness are often seen as complementary; in fact, researchers have used at least two common procedures to assess typicality/distinctiveness. First, in a deviation-based version, participants are asked to rate how much a specific face deviates in appearance from other known faces (DEV; e.g., [9]). In a second version, participants are asked to rate how easily they believe they would spot a face in a large group of people (face in the crowd, FITC, e.g., [10]). Although a common assumption is that both measures tap into the same underlying construct, recent research suggests that deviation-based and FITC-based assessments of typicality/distinctiveness may exhibit systematic differences [11]. In the present study, we therefore used both types of ratings.

We also consider empirical evidence suggesting that the representations of familiar as compared to unfamiliar faces are qualitatively different [12, 13]. More specifically, familiarity of a face might affect its perceived typicality in two different ways. First, it seems possible that familiar faces are perceived as more typical than unfamiliar faces: Assuming that the nMDFS is formed and recalibrated due to personal experience, familiar faces are frequently encountered, and thus might be expected to be perceived as more typical, and closer to the norm than unfamiliar faces. In fact, perceived typicality and perceived familiarity are highly correlated for artificial and natural categories [14, 15] and prototypical exemplars are perceived as more familiar [16]. Thereby, the norm has the potential to recalibrate—even in an experimental setting—due to adaptation to exemplars sharing the same untypical characteristic(s). Following adaptation, such characteristics can become perceptually more typical (e.g., [17–21]), and these effects can be relatively long lasting [22]. In sum, these adaptation studies indicate that familiarization to groups of faces with specific shared physiognomic features has the potential to increase the typicality of faces. Similarly, familiarity with a single face and its physiognomy could potentially enhance its typicality level. We call this the *recalibration hypothesis*, which would implicate that familiar faces would be perceived as more typical than their unfamiliar anti-faces.

By contrast, a second possibility is that familiar faces are perceived as more distinctive than unfamiliar faces. On the one hand, it may be unlikely that the real-life familiarization with some faces would substantially recalibrate the norm, to the extent that known faces are visually dissimilar and their representations are therefore broadly distributed in face space. On the other hand, recognition studies suggest that the representations for familiar faces are more robust and image-independent than those for unfamiliar ones [12, 23, 24]. For instance, familiar faces are still recognized well for low-quality images [25], or across large age difference or differences in viewpoint [26]. Similarly, personally familiar faces can be identified across a range of viewing distances [27] and discriminated using broader spatial frequency bands than unfamiliar faces [28]. In contrast, the processing of identity information from unfamiliar faces is rather poor [13], even in seemingly simple tasks such as matching a target face with eight simultaneously presented other identities [29]. These differences might be interpreted in terms of more distinctive mental representations for familiar compared to unfamiliar faces. A neurophysiological correlate of differences in representational quality between familiar and unfamiliar faces is a more negative N250 event related potential (ERP) component, with a typical onset around 200 ms, for famous faces compared to unfamiliar ones (e.g., [30–33]). Similarly, in a go/no-go paradigm, the earliest differences between personally familiar faces and unfamiliar faces in ERPs have been reported to occur from 210 ms post stimulus onset onwards [34]. A study on saccadic response times suggested even earlier recognition of personally familiar faces around 180 ms [35].

The finding that familiar faces can be more easily discriminated and recognized than unfamiliar faces could indicate increased resolution in the area of a familiar face in face space. This would implicate that the representational distance between two given faces A and B is enhanced when those faces are familiar, compared to when they are unfamiliar. Of relevance, recent research suggests that such differences could be related to the processing of holistic information in particular, rather than to processing of subtle local information in a face [36]. In the present paper, we refer to this notion of enhanced representation for familiar faces as the *superior representation hypothesis*. Such a different resolution could distort similarity or typicality ratings of familiar faces within the nMDFS, and increase their perceptual distance to the norm. For instance, Tanaka and colleagues [37] showed that a 50/50 percent morph of a typical and a distinctive face has a stronger perceptual resemblance to the distinctive face parent than the typical face parent. Based on these findings, they suggested that distinctive faces, which are located further away from the norm, also possess broader attractor fields in face space compared with more typical faces. Similarly, to the extent that a familiar face is perceived as further away from the norm than its (unfamiliar) anti-face, we should observe lower deviation-based typicality ratings for the familiar face than for its anti-face.

In addition to typicality/distinctiveness, we also assessed other more social judgements of faces. Note that the location of a face in nMDFS is not only defined by its distance to the norm (which may be of prime importance for typicality/distinctiveness), but its specific *vector direction* as well, which is determined by the specific relation of the dimensions to each other. While the vector direction may have little influence on perceived typicality, when measured as deviation from the norm (DEV; e.g., [9]), it could be crucial for more social judgements such as attractiveness, likability or trustworthiness. For example, enhanced compared to average levels of sexual dimorphism can be attractive for female faces [38]. Such an importance of the vector direction is highlighted in the statistical model of facial attractiveness [39].

Possible relations between typicality (or averageness) on the one hand, and likability and attractiveness on the other hand, have been investigated in previous research on the perception of objects and faces (e.g., [18, 40–42]). Studies on faces suggest a medium to large effect of typicality on attractiveness [43]. More typical faces are perceived as more familiar [14], and are attributed with more positive characteristics (likability, attractiveness) [14, 44, 45]. Typical faces may also be processed more fluently [46], and thus more efficiently, as a result of the larger number of category-common characteristics of more typical exemplars. Other studies demonstrated that adaptation to manipulated faces or objects is accompanied by changes in typicality and attractiveness [47] or likability [17, 18]. Such results led to the averageness hypotheses, according to which more typical or average exemplars are perceived as more attractive [48].

However, other studies casted doubt on a straightforward relation between typicality and attractiveness [49]. For example, Perrett and colleagues [50] showed that the average of 60 faces was less attractive than the average of the fifteen most attractive faces of the set. In particular, DeBruine et al [51] suggested that there is a dimension in face space that differentiates faces regarding their attractiveness and that this dimension would be independent of effects of averageness. Emerging from this debate, a statistical model of facial attractiveness [39] accounted for effects of *vector direction* as well. Here, faces were differentiated via 25 shape and 25 reflectance dimensions. Importantly, faces were perceived as most attractive for average weights assigned to some of these 50 dimensions on the average level, highlighting the importance of the distance to the norm. By contrast, faces were perceived as most attractive for more extreme weights assigned to some other dimensions, thus emphasizing a role for vector direction.

Finally, the relation between trustworthiness and typicality has also been studied [52]. Distance-to-norm has been found to be an important predictor of trustworthiness [53], in the sense that perceived trustworthiness decreases with increasing distance from the average face. Also, familiar faces, which lead to an increased feeling of safety [54] are likely to be perceived as more trustworthy than unfamiliar faces. However, since perceived trustworthiness, just like attractiveness, is additionally influenced by perceived sexual dimorphism [55], we consider the possibility that trustworthiness might be influenced by vector direction as well.

In the present study we tested whether faces and their corresponding anti-faces, which inherit the same physical distances to the norm, also share the same *psychological* distance. We assessed this relation for familiar and unfamiliar celebrity faces via rated levels of typicality (deviation-based, DEV), distinctiveness (face in the crowd, FITC), attractiveness, likeability and trustworthiness. Note that we assessed typicality/distinctiveness using two frequently used operationalisations: (1) typicality as the deviation to the norm (DEV; see also [9, 11]) and (2) distinctiveness via the face in the crowd rating (FITC, e.g., [10]). In line with the predictions of the nMDFS, we hypothesized that unfamiliar faces and their corresponding anti-faces would exhibit the same level of perceived typicality (DEV). For familiar faces, however, we assumed that faces and their corresponding anti-faces would differ in perceived typicality. Specifically, to the extent that the *recalibration hypothesis* holds true, familiar faces should be perceived as more typical than their anti-faces. By contrast, to the extent that the *superior representation hypothesis* holds true, familiar faces should be perceived as less typical than their anti-faces.

In line with recognition studies, we also expected that familiar faces should be rated as more distinctive than their unfamiliar anti-faces in a face-in-the-crowd assessment (FITC, e.g., [10]). For unfamiliar faces and their anti-faces we did not expect similar differences in distinctiveness (FITC). Finally, to the extent that the more social judgements (attractiveness, likability and attractiveness) correlate with typicality one would expect that the same physical distance would lead to equivalent judgements for unfamiliar faces and their anti-faces. However, to the extent that vector directions (e.g., the relationship between high levels of sexual dimorphism and perceived attractiveness) are important for a specific judgement, this would not necessarily be the case. For instance, it is plausible that celebrity faces tend to be of more than average attractiveness, and therefore will be perceived as more attractive than their anti-faces. Moreover, we considered that familiarity potentially triggers higher likability and trustworthiness ratings for familiar faces than their anti-faces [54].

## Material and Methods

### Participants

Data from 24 participants, all students at Friedrich Schiller University of Jena, Germany, contributed to this study (7 male; *M*_age_ = 22.0 years, *SD*_age_ = 2.5, range 19–30). All participants gave written informed consent, reported normal or corrected to normal vision and were compensated with course credit. The research reported in this study was performed in accordance with the Declaration of Helsinki, and was approved by the Faculty Ethics Committee of the Faculty for Social and Behavioral Sciences at the Friedrich Schiller University, Jena (Approval Number 13/07). Five additional participants (of the originally 30 participants) were excluded from data analyses, since they were not able to identify all of the international celebrities that were included in the stimulus set. One additional participant was excluded, because it turned out after the experiment that he had been previously living in Austria for a period of two years and thus had likely been exposed to the Austrian celebrity faces.

### Stimulus Material

#### Pilot study

A pilot study served to select suitable international and Austrian celebrities and to validate their familiarity and unfamiliarity for German participants. We decided to use unfamiliar national (Austrian) celebrities for the unfamiliar face category and familiar international celebrities (World) for the familiar face category, in order to better control for potential general category differences between unfamiliar and celebrity faces that might be irrespective of familiarity. Twelve participants from the Friedrich Schiller University of Jena, Germany (3 male; Mage = 22.6 years, SDage = 1.3, range 20–25), different to the ones described above, rated the familiarity of a total of eighteen male celebrities (nine Austrian: Armin Assinger, Alfred Dorfer, Alfons Haider, Felix Baumgartner, Gery Keszler, Karl Markovics, Rainhard Fendrich, Tobias Moretti und Werner Schreyer, and nine international: Brad Pitt, David Beckham, John Travolta, Kevin Costner, Leonardo DiCaprio, Nicolas Cage, Orlando Bloom, Robert Pattinson und Wladimir Klitschko). Participants completed three ratings: (1) they indicated on a four-point scale how well they knew the depicted person (0 = unknown, 1 = hardly known, 2 = known, and 3 = well-known), (2) they were asked to recall the name, and (3) they should provide semantic information that specified the source of fame for a given person (e.g., profession, specific song or movie, partner etc.). We compiled an overall score for familiarity with a maximum of 5 points: 0–3 points were assigned for question 1, and up to 1 point for question 2 and 3 each. Based on these ratings, the Austrian celebrities Alfred Dorfer, Alfons Haider, Felix Baumgartner, and Werner Schreyer were selected due to their low familiarity ratings (Mfamiliarity = 0.40, SDfamiliarity = 0.47), and David Beckham, Leonardo DiCaprio, John Travolta, and Nicolas Cage were selected due to their high levels of familiarity (Mfamiliarity = 4.71, SDfamiliarity = 0.30). Unfamiliar and familiar face categories were matched for profession and age. Each category consisted of three actors/models and one sportsman. Ages ranged from 40 to 60 years, and the mean age of the unfamiliar identities was 51 (SD = 6.29) and the mean age of the familiar identities was 49 (SD = 9.95).

#### Main experiment

Thus, the stimulus material was based on the images of eight male celebrities: four Austrian celebrities for the unfamiliar and four international celebrities for the familiar faces. The final stimuli consisted of (1) eight within-identity averages (averages across 20 different images of the same person) of these celebrity faces, henceforth referred to as “original identity” faces (OF), (2) the anti-faces (AF) of these eight original faces, and (3) an across-identity average face (AV) (average across 20 individual images of celebrities, with one image per international and Austrian celebrity, respectively).

During stimulus creation for the experiment great care was taken to avoid differences in general or low level image properties between the familiar and unfamiliar face categories, and between the original and the anti-faces. Such differences might occur during anti-face generation on the bases of different numbers of individual images contributing to the original inputs for a morph trajectory (i.e. an average face being based on more images than an individual face). We avoided such artefacts by using, both for the across-identity average face (AV) and for each within-identity original face average (OF) morphs across 20 images throughout. Averages were generated with GMorph software [23], using 82 facial landmarks. Anti-faces were then created by extrapolating between each ID and the average face, transforming both shape and reflectance (referenced to as “texture” in GMorph) information, to such an extent that all anti-faces had an “identity strength” of -100%. For graphical examples of the methods and resulting stimuli used in the present research, please refer to Faerber, Kaufmann, and Schweinberger [7] and Schweinberger and Schneider [56]. Minor image artefact corrections were applied to two anti-faces: in one case (A. Dorfer) the artefact was caused by fringes in the OF, in the second case (W. Schreyer) the artefact was caused by colour correction to a number of images contributing to the OF. All faces were standardized for height and images were resized to 380 x 570 pixels.

### Procedure

The experiment consisted of seven blocks which were presented in fixed order. In Block 1 participants previewed all face images, which were shown for 800 ms each in randomized order, with an inter-stimulus interval of 500 ms. In Blocks 2–6 participants rated the stimulus material on 6-point Likert scales regarding attractiveness (1 = very unattractive and 6 = very attractive), typicality (deviation-based; 1 = very untypical and 6 = very typical), distinctiveness (“face-in-the-crowd”; 1 = very undistinctive and 6 = very distinctive), likability (1 = very unlikable and 6 = very likable [in German “sehr unsympathisch” to “sehr sympathisch”]), and trustworthiness (1 = very untrustworthy and 6 = very trustworthy). Stimuli were presented in randomized order and termination of each image was self-paced via the rating keypress. Results from Block 7 (involving pairwise similarity ratings between stimuli) were unrelated to the main purpose of the present paper, and thus are not reported in detail below). In a post experimental questionnaire demographic questions were asked. In addition, the familiarity with the depicted faces was assessed post-experimentally, in exactly the same way as in the Pilot study. The total duration of the experiment was about 30 minutes.

## Results

**Fig 1:**
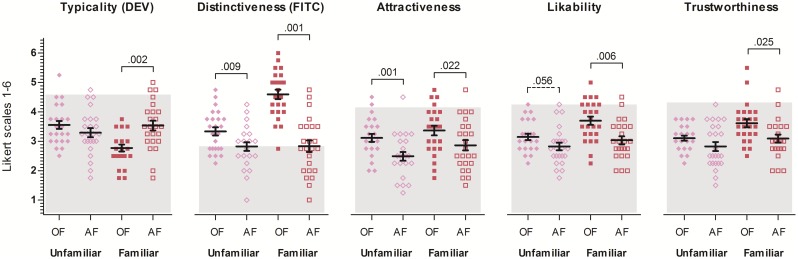
Results. Typicality, distinctiveness, attractiveness, likability, and trustworthiness ratings split by familiarity (unfamiliar and familiar) and by face polarity (original face, OF, and anti-face, AF). Means are accompanied by error bars indicating +1 standard error of the mean. Significant post-hoc simple mean comparisons for each comparison between OF and corresponding AF are marked with solid lines (significances) as well as dotted lines (trends) and *p*-values are indicated. Grey bars indicate the mean rating of the average face (typicality [*M* = 4.58, *SD* = 1.02], distinctiveness [*M* = 2.88, *SD* = 1.42], attractiveness [*M* = 4.13, *SD* = 0.95], likability [*M* = 4.21, *SD* = 0.98], and trustworthiness [*M* = 4.25, *SD* = 0.79].

An exploratory analysis revealed significant pairwise correlations between the rating variables: typicality and distinctiveness with *r*(16) = -.57, *p* = .023, attractiveness and sympathy with *r*(16) = .78, *p* < .001, attractiveness and trustworthiness with *r*(16) = .63, *p* = .009, as well as sympathy and trustworthiness with *r*(16) = .96, *p* < .001. Accordingly, we calculated an initial multivariate analysis of variance (MANOVA) with repeated measures on face polarity (original face: OF, vs. anti-face: AF) and familiarity (unfamiliar vs. familiar), and typicality, distinctiveness, attractiveness, likability and trustworthiness as dependent variables. Results showed a strong overall effect for face polarity, *F*(1,19) = 10.91, *p* < .001, ηp^2^ = .74, familiarity, *F*(1,19) = 11.77, *p* < .001, ηp^2^ = .76, and their interaction, *F*(1,19) = 9.09, *p* < .001, ηp^2^ = .71. Please refer to Table 1 and Fig 1 for a more detailed overview of the results.

**Table 1:** Main effects and interactions of the MANOVA with the repeated measurement factors face polarity (FP; original face vs. anti-face), and familiarity (Fam; unfamiliar vs. familiar) and the dependent variables typicality, distinctiveness, attractiveness, likability and trustworthiness.

	Typicality (deviation based)			Distinctiveness (face in the crowd)			Attractiveness			Likability			Trustworthiness		
	*F*_(1,23)_	*P*	ηp^2^	*F*_(1,23)_	*p*	ηp^2^	*F*_(1,23)_	*p*	ηp^2^	*F*_(1,23)_	*p*	ηp^2^	*F*_(1,23)_	*p*	ηp^2^
FP	2.50	*n*.*s*.	---	39.07	< .001	.63	11.82	.002	.34	7.76	.011	.25	4.93	.036	.18
Fam	10.15	.004	.31	39.42	< .001	.63	6.37	.019	.22	22.31	< .001	.49	22.04	< .001	.49
FP × Fam	17.05	< .001	.43	24.56	< .001	.52	<1	*n*.*s*.	---	5.88	.024	.20	2.87	*n*.*s*.	---

### Typicality (DEV)

The results showed a significant effect of familiarity, as well as a significant interaction between face polarity and familiarity (Table 1). Importantly, post-hoc *t*-tests demonstrated that unfamiliar original faces did not differ significantly in typicality from their anti-faces, *t*(1,23) = 1.39, *p* = .177, *n*.*s*. In contrast, there was a significant difference between typicality ratings for familiar original faces and their anti-faces, *t*(1,23) = 3.56, *p* = .002, *d* = 1.08, with lower typicality ratings for familiar faces than for their corresponding anti-faces. There was also a significant difference between typicality ratings for familiar original faces and unfamiliar original faces, *t*(1,23) = 4.71, *p* < .001, *d* = 1.27, with lower typicality ratings for familiar than unfamiliar original faces.

### Distinctiveness (FITC)

For distinctiveness ratings we found significant effects of face polarity, familiarity and their interaction (Table 1). Post-hoc *t*-tests revealed that both—unfamiliar and familiar—OFs were rated as more distinctive than their anti-faces, *t*(1,23) = 2.86, *p* = .009, *d* = 0.72 and *t*(1,23) = 6.85, *p* < .001, *d* = 2.00, respectively. However, the interaction reflects the fact that this effect was larger for the familiar condition; familiar OFs were perceived as most distinctive of all face “classes” (see Fig 1 and Table 2). There was also a significant difference between distinctiveness ratings for familiar original faces and unfamiliar original faces, *t*(1,23) = 7.93, *p* < .001, *d* = 1.69, with higher distinctiveness ratings for familiar than unfamiliar original faces.

**Table 2:** Top: Main effects of ANOVAs with repeated measurements on the factor face class (familiar original faces [fam. OF] vs. unfam. OFs vs. anti-faces (AF) of familiar faces vs. anti-faces of unfamiliar faces vs. average face) for the dependent variables typicality (deviation based, DEV), distinctiveness (face in the crowd, FITC), attractiveness, likability, and trustworthiness. Below: Post-hoc comparisons of familiar or unfamiliar OFs and familiar or unfamiliar AFs with the average face ratings.

	Typicality (deviation based)			Distinctiveness (face in the crowd)			Attractiveness			Likability			Trustworthiness		
	ANOVAs														
	*F*_(1,23)_	*p*	ηp^2^	*F*_(1,23)_	*p*	ηp^2^	*F*_(1,23)_	*p*	ηp^2^	*F*_(1,23)_	*p*	ηp^2^	*F*_(1,23)_	*p*	ηp^2^
FC	**22.04**	**< .001**	**.49**	**17.41**	**< .001**	**.43**	**19.44**	**< .001**	**.46**	**18.07**	**< .001**	**.44**	**18.23**	**< .001**	**.44**
	*T*-tests														
	*T*_(23)_	*p*	*d*	*T*_(23)_	*p*	*d*	*T*_(23)_	*p*	*d*	*T*_(23)_	*p*	*d*	*T*_(23)_	*p*	*d*
Fam. OFs	**7.42**	**< .001**	**2.19**	**-5.06**	**< .001**	**1.48**	**3.79**	**.001**	**.87**	**2.32**	**.030**	**.60**	**2.85**	**.009**	**.87**
Unfam. OFs	**4.98**	**< .001**	**1.20**	-1.58	*n*.*s*.	---	**4.62**	**< .001**	**1.24**	**5.46**	**< .001**	**1.35**	**7.26**	**< .001**	**1.82**
Fam. AFs	**4.38**	**< .001**	**1.14**	< 1	*n*.*s*.	---	**5.89**	**< .001**	**1.39**	**5.20**	**< .001**	**1.41**	**5.31**	**< .001**	**1.59**
Unfam. AFs	**6.11**	**< .001**	**1.43**	< 1	*n*.*s*.	---	**7.09**	**< .001**	**1.94**	**6.72**	**< .001**	**1.68**	**6.33**	**< .001**	**1.86**

*Note*. In all variables face classes differed significantly from the average face apart from distinctiveness. Here, only familiar OFs differed significantly and were rated most distinct (face in the crowed measure). Remarkably, while familiar OFs earned contrastive ratings compared to the average for typicality (DEV) and distinctiveness (FITC), they were rated second highest after the average face in more social variables attractiveness, likability and trustworthiness.

### Social variables

#### Attractiveness

The analyses revealed significant main effects of face polarity and familiarity, but no interaction. Familiar faces were rated as more attractive overall, and both unfamiliar and familiar OFs were rated significantly more attractive than their corresponding AFs: for unfamiliar faces, *t*(1,23) = 3.73, *p* = .001, *d* = 0.91 and for familiar faces, *t*(1,23) = 2.46, *p* = .022, *d* = 0.60.

#### Likability

Main effects for face polarity and familiarity, and their interaction reached significance. Importantly, *t*-tests showed that unfamiliar OFs and their corresponding AFs did not significantly differ in likability, *t*(1,23) = 2.01, *p* = .056, *d* = 0.55, whereas familiar OFs and their AFs did, *t*(1,23) = 3.06, *p* = .006, *d* = 0.99. Thus, familiar faces were rated more likable than their anti-faces, while unfamiliar faces and their anti-faces did not differ. There was also a significant difference between likability ratings for familiar original faces and unfamiliar original faces, *t*(1,23) = 4.59, *p* < .001, *d* = 0.90, with higher likability ratings for familiar than unfamiliar original faces.

#### Trustworthiness

Here, we found only significant main effects of face polarity and familiarity. In short, OFs were rated as somewhat more trustworthy than AFs, and faces in the familiar condition were rated as more trustworthy than in the unfamiliar condition. The interaction between those factors did not reach significance. However, separate *t*-tests indicated that whereas familiar OFs were rated significantly more trustworthy than their AFs, *t*(1,23) = 2.40, *p* = .025, *d* = 0.78, this difference was not significant for unfamiliar faces, *t*(1,23) = 1.68, *p* = .106, *n*.*s*. Finally, there was also a significant difference between trustworthiness ratings for familiar original faces and unfamiliar original faces, *t*(1,23) = 4.36, *p* < .001, *d* = .91, with higher trustworthiness ratings for familiar than unfamiliar original faces.

### Relation to the Norm (Average Face)

We were additionally interested in how the different face classes used in this study (unfamiliar OF, unfamiliar AF, familiar OF, familiar AF) would be perceived in relation to the average face. Therefore, we calculated additional ANOVAs with face class (unfamiliar OF, unfamiliar AF familiar OF, familiar AF, and AV) as within-subject factor for each of the dependent variables described above, and further tested each face class against the average face in case of a significant main effect (Table 2).

Unsurprisingly, all ANOVAs showed significant main effects of face class. Moreover, post-hoc *t*-tests revealed significant differences between the average face and all other face classes for all variables with the exception of distinctiveness. For distinctiveness, familiar original faces were the only class that was rated as significantly more distinctive than the average face (Table 2 and also Fig 1). It may be noted that for both typicality and distinctiveness, ratings for familiar original faces deviated most from the average face, compared with all other face classes (see Fig 1). By contrast, for ratings on all social variables (attractiveness, likability, trustworthiness), the average face earned the most favourable ratings, followed by familiar original faces.

### Control Experiment

Despite our efforts into ensuring that both familiar and unfamiliar faces are well matched with respect to sex, age, occupation, and general appearance, an inherent limitation of the present study design that it is difficult to address is the concern that there is something special about the particular internationally famous faces used in the familiar face condition, as compared to the nationally famous faces that were unfamiliar for our participants in the main experiment. For that reason, we focussed on comparing ratings for familiar and unfamiliar faces with respect to the difference between original and anti-face versions (rather than on comparing ratings for original familiar and unfamiliar faces directly). However, a control experiment explicitly addressed the degree to which original face stimuli in the two sets were well matched for the variables under investigation. While an ideal option to assess any stimulus differences between our familiar internationally famous faces and our unfamiliar nationally famous faces would be to test participants who are unfamiliar with all faces, we were unable to identify suitable participants unfamiliar with the international celebrities. Instead, we tested a group of participants living in Austria, who were all familiar with both the internationally famous and the nationally famous celebrities.

Eighteen participants were tested at the University of Vienna, Austria (2 male; *M*_age_ = 29.3 years, *SD*_age_ = 7.2, range 18–39), who were well familiar with all the identities in both face classes (internationally famous and nationally famous). Stimulus material and procedure was identical to that used in the main experiment. In data analysis, we focused on the comparison of the two original face classes (internationally famous versus nationally famous).

Importantly, internationally famous and nationally famous familiar original faces received similar ratings for typicality, *M* = 3.82 and 3.99, respectively, *t*(1,17) = 1.16, *p* = .264, *d* = 0.15. At the same time, internationally famous original faces received higher ratings than nationally famous faces on distinctiveness, *M* = 4.88 and 4.00, respectively, *t*(1,17) = 6.91, *p* = .001, *d* = 1.02. Regarding social variables, internationally famous original faces and nationally famous faces received similar ratings of attractiveness, *M* = 3.56 and 3.47, respectively, *t*(1,17) = 0.50, *p* = .621, *d* = 0.13. Similarly, these face classes did not differ with respect to both rated likability, *M* = 3.83 and 3.86, respectively, *t*(1,17) = 0.16, *p* = .872, *d* = 0.04, and rated trustworthiness, *M* = 3.56 and 3.79, respectively, *t*(1,17) = 1.42, *p* = .173, *d* = 0.32.

In the main experiment, deviation-based typicality of familiar faces had been rated as significantly lower compared to unfamiliar faces. Here we show that deviation-based typicality of these face classes was equivalent for participants who were familiar with the faces in both classes. Accordingly, we may conclude that the two face classes were well matched for deviation-based typicality. With respect to distinctiveness (face in the crowd, FITC-rating), the results are less clear, as we found higher rated distinctiveness for internationally famous compared with nationally famous celebrities, in this sample of Austrian participants as well. One possible explanation of this difference is based on earlier findings that FITC-based ratings of distinctiveness may be particularly susceptible to cognitive heuristics. For instance, it seems plausible to assume that, following explicit recognition of a face as an international “superstar”, participants are biased towards exaggerated ratings of how easily they would spot that face in a crowd. In any case, the combined results for deviation-based ratings of typicality and FITC-based ratings of distinctiveness provide additional support for the idea that these two measures assess at least partially different constructs, and are broadly in line with the suggestion that FITC ratings are more easily biased by cognitive heuristics[11].

Finally, with respect to ratings on the social variables of rated attractiveness, likability, and trustworthiness, there was no evidence for differences between internationally famous and nationally famous celebrities in the Austrian participant sample. This is in clear contrast to the findings from German participants in the main experiment who were unfamiliar with the nationally famous Austrian celebrities, and who produced significantly lower ratings of attractiveness, likability, and trustworthiness for this face class. Accordingly, we may conclude that the differences in social variables in the main experiment can be safely attributed to the familiarity of faces per se, rather than to any subtle visual differences between the face classes.

## Discussion

To the best of our knowledge, the present study is the first to demonstrate that familiar faces were rated differently from their anti-faces on all five assessed variables typicality, distinctiveness, attractiveness, likability, and trustworthiness. This is in strong contrast to the idea that the distance to the norm is the only factor that influences perceived typicality (DEV) and distinctiveness (FITC), as predicted by the norm-based MDFS model. Instead, our results are generally consistent with the assertion that the mental representations of familiar and unfamiliar faces differ. In particular, familiar faces (but not their anti-faces, nor unfamiliar faces) were perceived as highly untypical (DEV) and highly distinctive (FITC). These results can be reconciled with face recognition studies, which demonstrated differences in representational quality between familiar and unfamiliar faces, with more robust representations for familiar faces [12, 23, 24]. Our results are also compatible with the *superior representation hypothesis* as stated in the introduction, while they are at variance with the *recalibration hypothesis*. It is particularly hard to reconcile with the recalibration hypothesis that differences in rated typicality and distinctiveness (compared to the average face), rather than being small, were in fact most pronounced for original familiar faces.

Importantly, the perceived typicality (DEV) of unfamiliar faces and their anti-faces was equivalent, in line with predictions from the nMDFS model. Overall, the prediction of this model that faces and their anti-faces have equivalent distances to the norm could be confirmed for unfamiliar faces, but not for familiar ones. With respect to distinctiveness (FITC), although the data partly looked complementary to those for typicality (see Fig 1), the pattern of findings was less straightforward. In particular, while higher distinctiveness (FITC) ratings for familiar OFs compared to their AFs also appear to be consistent with the superior representation hypothesis, the smaller but significant difference between unfamiliar OFs compared to their AFs was not predicted. Although it is difficult to exclude the possibility that this result might partly reflect residual familiarity of our participants with Austrian national celebrities, the evaluation of a post-experimental assessment of familiarity with the stimulus faces provided little support for this idea: First, no single face in the unfamiliar condition could be identified by any of our participants. Second, post-experimental familiarity ratings (unfamiliar faces: *M* = 0.90, *SD* = 0.55; familiar faces: *M* = 4.90, *SD* = 0.23, five-point scale from 0 to 5) confirm very low ratings for unfamiliar faces. Nevertheless, it may be noted that post-experimental familiarity ratings appeared slightly inflated, when compared to familiarity ratings of the same stimuli by naïve observers (see pilot study). Note that post-experimental ratings were obtained after all faces had been seen many times. Because it is well-known that repeated presentation causes a subjective feeling of familiarity (e.g., [57]); we believe it is highly likely that repeated presentation during the experiment will have slightly inflated our post-experimental familiarity ratings. Of relevance, other studies suggested that FITC measures of distinctiveness could be additionally susceptible to biases from cognitive heuristics. For instance, when asked how likely they would notice a face in a crowd, participants might confuse distinctiveness judgements with a tendency to favour attractive faces (“Surely I would notice such an attractive face”, see [11]). In our view, this may be a more likely explanation for the fact that both familiar and unfamiliar original faces were judged more distinctive than their anti-face counterparts. In this context, it seems noteworthy that attractiveness ratings were the only other rating variable that exhibited this parallel pattern of findings as seen in FITC ratings.

The fact that both distinctiveness and attractiveness ratings were higher for unfamiliar OFs than their AFs is inconsistent with an account that is purely based on distance-to-norm. Instead, it seems that vector direction is relatively important for those two ratings in particular. While our study was not designed to reveal the relative roles of specific dimensions of facial variation, it should be noted that vector direction (e.g., for sexual dimorphism, see [38]) over and above distance-to-norm has been assumed to influence ratings of facial attractiveness [39]. To the extent that the present celebrity faces from both familiarity conditions tend to be of more than average attractiveness, our findings can be reconciled with Said and Todorov’s [39] model of facial attractiveness.

Finally, the social judgements of likability and trustworthiness, just like typicality judgements, were comparable for unfamiliar OFs and their AFs. At the same time, familiar OFs obtained higher likability and trustworthiness ratings than their AFs, despite the fact that the interaction between face polarity and familiarity did not reach significance in the case of trustworthiness. These results are in line with the hypothesis that familiarity breeds likability and trustworthiness. Ratings for unfamiliar faces and their anti-faces were equivalent, as predicted by their distance to the norm. Moreover, among all original faces and anti-faces, familiar original faces received the highest ratings for likability and trustworthiness (although these were significantly lower compared with the average face which received the highest ratings). These results are reminiscent of previous reports of an association of these variables with averageness. For example, Sofer et al. [52] showed a positive relation between distance-to-norm and trustworthiness. Despite the fact that our findings are generally in line with this earlier research, it needs to be acknowledged that the recognition of a celebrity invokes the activation of conceptual and semantic information about a person, which in turn can exert an effect on evaluative ratings such as for likability and trustworthiness. Thus, more research is needed to investigate the relative contributions of visual familiarity and semantic information for ratings of likability or trustworthiness for faces.

In sum, the present study showed that an average face received the highest ratings for typicality, attractiveness, trustworthiness and likability, and the lowest ratings for distinctiveness. These results confirm earlier studies showing that prototypes are perceived as most typical and attractive [14]. The most important finding of the present study was that while typicality ratings for unfamiliar faces and their anti-faces were equivalent, in line with a distance-to-norm account, familiar faces were perceived as much less typical than their anti-faces, in line with a *superior representation hypothesis* for familiar faces. Compared to their anti-faces, familiar faces were also perceived as more distinctive, more attractive, more likable and more trustworthy. For unfamiliar faces, corresponding differences were only seen for ratings of distinctiveness and attractiveness. Overall, the present findings suggest (1) that familiarity needs to be considered in studies of mental representations of faces, and (2) that familiarity, distance-to-norm and vector direction make different and interactive contributions to different types of evaluations of faces.

## Supporting Information

S1 Table Data for main analyses (MANOVA). (XLSX)

S2 Table Data for analyses of section “Relation to the norm (average face). (XLSX)
